# Metal artefact reduction for accurate tumour delineation in radiotherapy

**DOI:** 10.1016/j.radonc.2017.09.029

**Published:** 2018-03

**Authors:** David Gergely Kovacs, Laura A. Rechner, Ane L. Appelt, Anne K. Berthelsen, Junia C. Costa, Jeppe Friborg, Gitte F. Persson, Jens Peter Bangsgaard, Lena Specht, Marianne C. Aznar

**Affiliations:** a Department of Oncology, Copenhagen University Hospital Rigshospitalet, Denmark; b Faculty of Health and Medical Sciences, University of Copenhagen, Denmark; c Biomedical Engineering, Department of Electrical Engineering, Technical University of Denmark, Lyngby, Denmark; d Niels Bohr Institute, University of Copenhagen, Denmark; e Leeds Institute of Cancer and Pathology, University of Leeds, and Leeds Cancer Centre, St. James’s University Hospital, UK; f Department of Clinical Physiology, Nuclear Medicine and PET, Rigshospitalet Copenhagen University Hospital, Denmark; g Department of Radiology, Copenhagen University Hospital Herlev Gentofte, Denmark; h Clinical Trial Service Unit, Nuffield Department of Population Health, University of Oxford, UK

**Keywords:** Dual energy CT, Iterative metal artefact reduction, Delineation uncertainty, IGRT

## Abstract

**Background and purpose:**

Two techniques for metal artefact reduction for computed tomography were studied in order to identify their impact on tumour delineation in radiotherapy.

**Materials and methods:**

Using specially designed phantoms containing metal implants (dental, spine and hip) as well as patient images, we investigated the impact of two methods for metal artefact reduction on (A) the size and severity of metal artefacts and the accuracy of Hounsfield Unit (HU) representation, (B) the visual impact of metal artefacts on image quality and (C) delineation accuracy. A metal artefact reduction algorithm (MAR) and two types of dual energy virtual monochromatic (DECT VM) reconstructions were used separately and in combination to identify the optimal technique for each implant site.

**Results:**

The artefact area and severity was reduced (by 48–76% and 58–79%, MAR and DECT VM respectively) and accurate Hounsfield-value representation was increased by 22–82%. For each energy, the observers preferred MAR over non-MAR reconstructions (*p* < 0.01 for dental and hip cases, *p* < 0.05 for the spine case). In addition, DECT VM was preferred for spine implants (*p* < 0.01). In all cases, techniques that improved target delineation significantly (*p* < 0.05) were identified.

**Conclusions:**

DECT VM and MAR techniques improve delineation accuracy and the optimal of reconstruction technique depends on the type of metal implant.

Modern advances in image-guided radiotherapy (IGRT) have increased the accuracy of dose delivery and decreased the need for large planning target volumes (PTV) [1]. The high level of accuracy in delivery has, however, increased requirements for accuracy in target delineation. Even deviations on a scale of a few millimetres may result in increased irradiation of organs at risk (OARs) or geographic target miss, and hence have significant negative impact on patient outcomes. Today, it is recognized that target and OAR delineation variability is a major source of uncertainty in radiotherapy (RT), and considerable work has been done in order to reduce factors that lead to this variability [2], [3], [4], [5], [6].

A major cause of delineation variability is streaking and beam hardening artefacts from metallic implants. Metallic artefacts are a significant clinical issue in RT, causing decreased delineation confidence, decreased dose calculation accuracy, and increased time spent on manually delineating pixels affected by the artefacts [7], [8], [9], [10], [11].

The two main methods described in the literature for reduction of metal artefacts are dual energy computed tomography (DECT) virtual monochromatic (VM) extrapolations [12] and iterative metal artefact reduction (MAR) algorithms [13], [14], [15]. DECT VM images between 95 and 150 kilo electron volt (keV) levels have been found to reduce beam hardening artefacts from various metallic prostheses effectively [16], [17], [18], while VM images around 40–70 keV show some clinical value by improving contrast to noise ratios (CNR) between soft tissues [19], [20]. MAR algorithms also show clinical value by reducing metal artefacts [21], [22] and improving dose calculation accuracy [23]. Using DECT and MAR in combination may enable a further reduction of artefacts [24].

In this work, we focused on the impact of metal artefact reduction on target delineation, hypothesizing that DECT and MAR techniques can reduce RT-specific uncertainties. We evaluated different combinations of DECT VM and MAR techniques both quantitatively and qualitatively in order to identify the optimal solutions for radiotherapy imaging in different anatomical regions. Furthermore, we investigated the potential of DECT VM and MAR to improve the accuracy of target delineation in patient and phantom images.

## Materials and methods

We compared six different reconstruction methods using dental, spine, and hip implant phantoms and corresponding patient cases. The six evaluated reconstructions were (1) 120 peak kilo voltage (kVp) (standard-of-care) (2) 120 kVp MAR (3) 70 keV DECT (4) 70 keV DECT MAR (5) 130 keV DECT and 6) 130 keV DECT MAR. Image acquisition details are described in the supplementary material. 70 and 130 keV levels were selected because they represent the mean energy of the polychromatic spectrum in a 120 kVp image while maximizing the CNR and the optimal balance between beam hardening artefact reduction and soft tissue contrast, respectively [17], [25].

These reconstructions were evaluated according to (A) the size/severity and HU-values of the artefacts, (B) the resulting image quality as evaluated by five observers and (C) the delineation variability from five observers.

### Phantoms

**Fig. 1:**
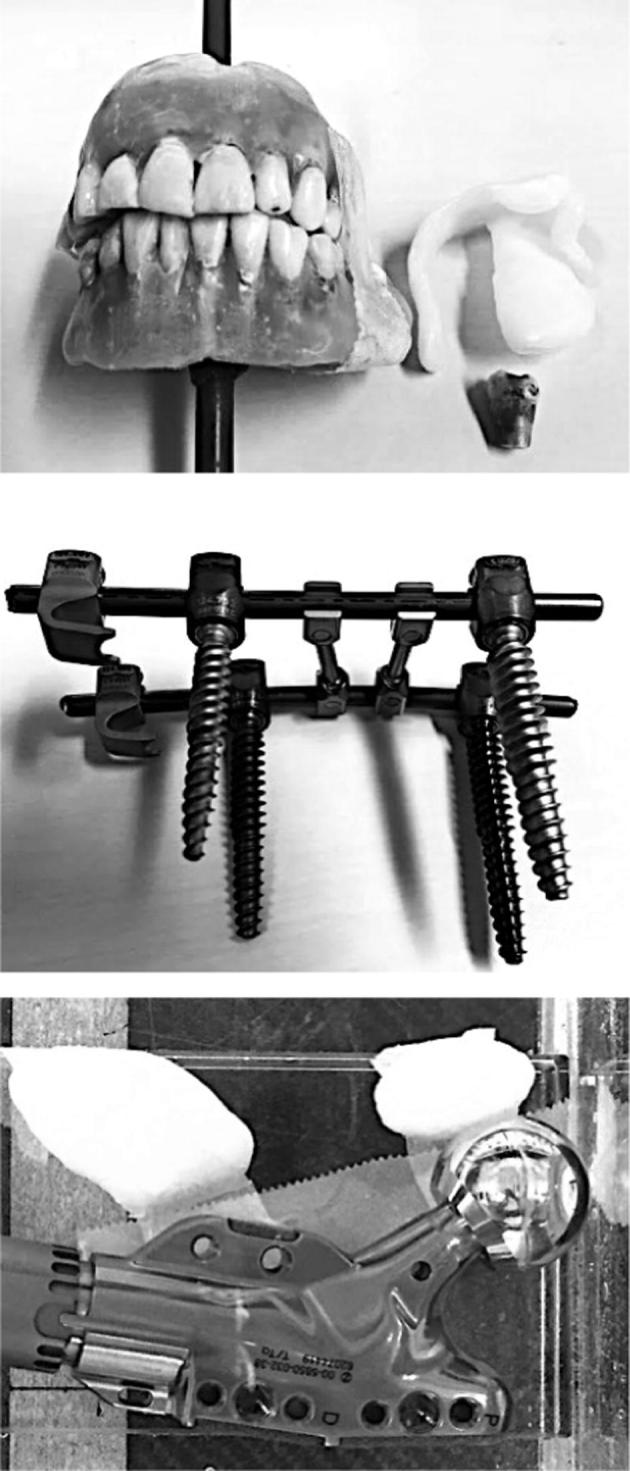
Phantoms used for quantitative analysis. Left: Dental phantom with low-contrast target removed for visibility (during the scan the low-contrast target was placed inside the oral cavity) and removable tooth with amalgam filling. Middle: Surgical Spine Screws. Right: Hip implant placed in water tank with two low-contrast targets.

Three phantoms (Fig. 1) with metal implants were constructed to represent common causes of metal artefacts as seen in clinical practice: (1) a set of human teeth fixated in paraffin wax with a removable amalgam-filled tooth (in-house construction) (2) surgical spine screws (Globus Revere Pedicle Screw System and K2M titanium rod) (3) a hip implant (Zimmer Segmental System Proximal Femoral Provisional and Femoral Head Provisional).

Low contrast targets were arbitrarily shaped of polycaprolactone (Polymorph, Thermoworx Ltd.) and placed in a water-tank together with the respective implants. The metal implants could be removed without affecting the positioning of the other objects in the tank. CT scans of each phantom were acquired with and without the presence of the metal implants.

### Patients

This study was approved by the institutional review board and the regional ethics committee (H-15006887). Patients who were referred for radiotherapy, had metal implants and were older than 50 years were offered inclusion. Informed consent was obtained for experimentation with human subjects. The DECT scans were performed immediately after the treatment planning CT scan (64-slice single-source CT scanner, Siemens Somatom Definition AS, Siemens Health Care, Forchheim Germany). Images were then reconstructed using the MAR algorithm (iMAR on VA48A SW, Siemens AG, München Germany). VM images were reconstructed using the Dual Energy application (syngo.CT Monoenergetic 2016, Siemens AG). Detailed settings and scanning procedure are described in the supplementary material (section “Image Acquisition” and Table A1).

### Study A: Artefact quantification

Phantom scans with and without metal present were acquired within one imaging session. The low-contrast targets and the surrounding water were segmented in the images without metal. These contours were then transferred to images with metal.

A program was written (MATLAB 2015b, MathWorks® Natick Massachusetts, U.S.A) to evaluate the area and severity of the artefacts, as well as the accuracy of HU-value representation of the water and low-contrast target within a region surrounding the metal implant.

The artefact area, HU-value median and interquartile range was calculated, where the latter was interpreted as an expression of artefact severity.

The 95% confidence intervals (CI) of the pixel intensity values of water and of the low-contrast target regions were estimated from the non-metal reference. If pixel intensity values in the image with metal were within the expected 95% CI for each corresponding region, the pixels were considered “accurately represented”. Pixels outside this range were considered “artefact”.

### Study B: qualitative assessment of patient images

The six reconstruction methods were evaluated by five observers (three oncologists and two radiologists) in CT scans of three patients with dental fillings, a spine implant, and a hip implant respectively. A program was developed in MATLAB for pairwise side-by-side comparison of the six reconstructions. The program presented the observers with all possible unique combination pairs of the six reconstructions in random order. Each combination was presented twice (first left to right, then right to left resulting in a total of 30 comparisons). By default, images were displayed in a standard soft-tissue window (centre 40 width 350). Observers were able to change the window setting and to simultaneously scroll through both reconstructions in order to evaluate each axial slice with metal. For each comparison, the observers were asked to choose the image they would prefer for RT delineation.

The comparison of a reconstruction to each of the five other reconstructions was considered an individual observation, and a preference for a reconstruction in the pairwise comparison was considered a positive outcome. A multivariate logistic regression analysis was conducted to identify factors (VM energy and/or use of MAR) significantly contributing to the probability of observers having a preference for a given reconstruction. Standard-of-care reconstructions (120 kVp) were considered the baseline, to which all other reconstructions (120 kVp MAR, 70 keV DECT, 70 keV DECT MAR, 130 keV DECT and 130 keV DECT MAR) were compared. Interaction between the factors (energy and use of MAR) was included in the analysis as well.

### Study C: delineation accuracy

Prior to delineation, the order of presentation of reconstructions was determined according to a ranking of the results of study (A) and (B). This was necessary to prevent information from previous reconstructions with better visibility influencing results on subsequent reconstructions. The images were presented to the five study observers in the determined order in standard delineation software used in our clinic (Eclipse version 13.6, Varian, Palo Alto, California, USA). The observers were blinded to the reconstruction type and delineation on the reference image was performed last.

In order to obtain the true contour of the low-contrast target, delineation was performed on a high-quality scan of the low-contrast target in air, and then transferred to the phantom images. A standard of care 120 kVp phantom scan without metal was included for reference. In order to provide metrics for both positional and volumetric differences between contours [26], we measured the maximal Hausdorff distance^1^ (HD) and the Dice coefficient^2^ (DC; both metrics measured in 3D slicer, open source software, BWH Harvard, https://www.slicer.org, [27]) for each observer’s contour relative to the true contour.

A Friedman-test was used for primary analysis and a pairwise post hoc Nemenyi-test analysis with correction for multiple testing was used to test for significant differences between contours performed by the study observers. All statistical analyses were performed in *R* version 3.2.2 (R Foundation for Statistical Computing, Vienna, Austria).

## Results

### Study A: Artefact quantification

**Fig. 2:**
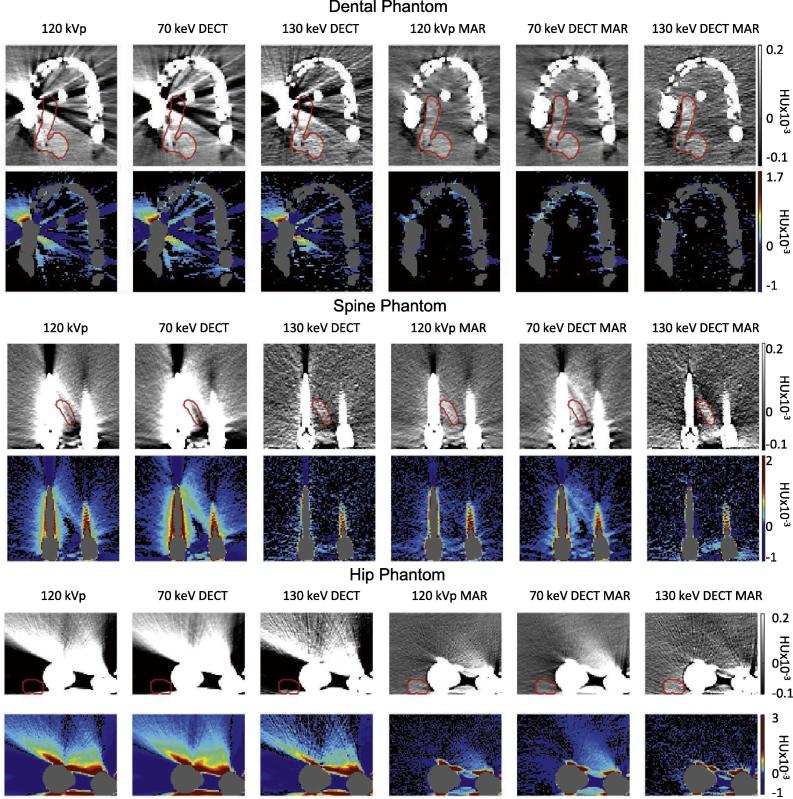
Dental (top), spine (middle) and hip (bottom) phantoms with metal implants shown in standard acquisition for radiotherapy treatment planning (left column) and in five reconstruction modalities. Odd rows: Un-manipulated CT reconstructions in a soft tissue window (centre: 40, width: 350). The red contour shows the true shape of the target as estimated from a scan without metal. Even rows: artefact–only images (water and tumour Hounsfield (HU) values, teeth and metal excluded from the calculation). Artefact pixels are shown on a colour scale while correctly-represented pixels are shown in black (no windowing was used). Notably, the iterative metal artefact reduction algorithm (MAR) reconstructions resulted in the highest number of correctly represented pixels in the dental and hip phantoms, while dual energy CT (DECT) virtual monochromatic reconstructions at the 130 keV level resulted in the highest number of correctly represented pixels in the spine phantom.

The six reconstructions of each phantom are presented in Fig. 2. Analysis was performed on the region shown for each phantom respectively. Artefact size, severity and the HU-value representation in the water and low-contrast target are presented in supplementary Table A2. The reduction in artefact area ranged between 48 and 76%, with the largest reduction found with 130 keV MAR for all phantoms.

*Dental filling phantom:* The largest reduction in artefact area (75%) and severity (70%) was measured using the 130 keV DECT MAR reconstruction. 120 kVp MAR resulted in the highest improvement in HU-value representation (increase from 73% to 97% in water and 59% to 97% in target), with no clear difference in results between the 120 kVp MAR and DECT MAR images.

*Spine implant phantom:* The largest reduction in artefact area was measured in the 130 keV DECT MAR reconstruction (48%). The lowest artefact severity was found with 120 kVp MAR (58%). 130 keV DECT resulted in the highest improvement in HU-value representation (increase from 30% to 79% in water and from 35% to 79% in target).

*Hip implant phantom:* The largest reduction in artefact area (60%) and severity (79%) was measured in the 130 keV DECT MAR image. 130 keV DECT MAR resulted in the highest improvement in HU-value representation (increase from 3% to 65% in water and 4% to 86% in target^3^).

### Study B: qualitative assessment of patient images

**Fig. 3:**
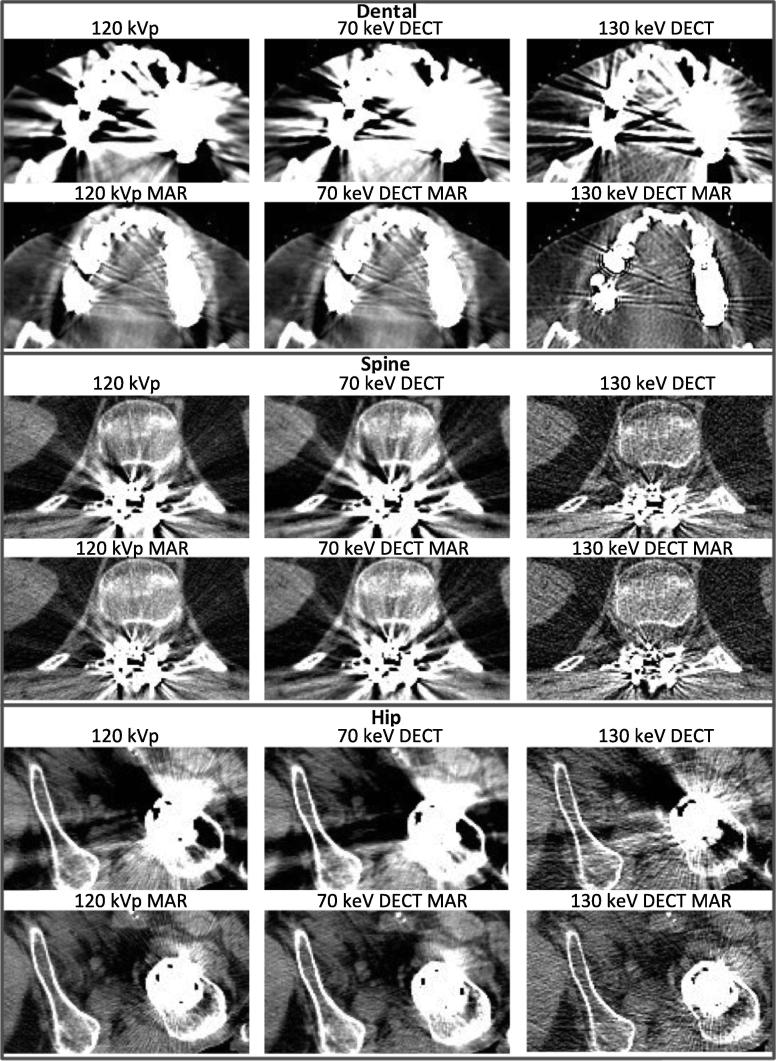
Three patients shown in standard acquisition scan for radiotherapy treatment planning (120 kVp) and in the five studied reconstruction modalities (70 keV DECT, 130 keV DECT, 120 kVp MAR, 70 keV DECT MAR and 130 keV DECT MAR). Two radiologists and three oncologists performed pairwise comparisons to rank the images. For contouring in the artefact affected area, observers preferred the combination of dual energy CT virtual monochromatic extrapolation at the 130 keV level and the iterative metal artefact reduction algorithm (130 keV DECT MAR) in the presence of artefacts from dental and spine implants, while 120 kVp MAR was preferred in the presence of artefacts from hip implants.

The six image reconstructions of each of the three patient scans are presented in Fig. 3. Table 1 shows which reconstructions were preferred by the observers as a percentage of the total number of times an image was presented. The most preferred reconstructions by count were 130 keV DECT MAR for dental and spine implants and 120 kVp MAR for the hip implant.

**Table 1:** Score (ratio of number of times displayed to number of times selected as the best image) of each reconstruction as evaluated visually by the observers. Each reconstruction was presented 50 times (10 times to each of the five observers).

Qualitative assessment of patient images: preferred reconstructions						
Patient Implant	120 kVp [%]	70 keV DECT [%]	130 keV DECT [%]	120 kVp MAR [%]	70 keV DECT MAR [%]	130 keV DECT MAR [%]
Dental	24	0	36	64	82	94
Spine	34	0	80	60	28	98
Hip	26	12	22	86	78	76

*Dental filling and hip implant patients:* The multivariate logistic regressions analysis showed that MAR had a statistically significant impact on the probability of observers preferring a given reconstruction (*p* < 0.01 for both), while the different VM energies showed no significant effect.

*Spine implant patient:* The same statistical analysis showed that 130 keV DECT VM energy as well as the MAR algorithm significantly enhanced the observers’ preference for selecting a given reconstruction (*p* < 0.01 and *p* < 0.05, respectively); however, no interaction between energy and the use of MAR was found.

### Study C: delineation accuracy

**Fig. 4:**
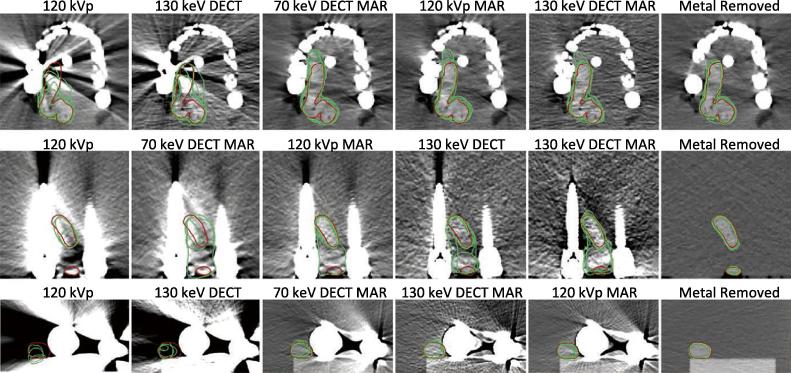
Low-contrast target delineation on phantom scans. Top row: dental fillings, middle row: spine screws, bottom row: hip implant. The rightmost column shows the scan with observer’s contours without metal in the setup. The remaining columns present the standard of care images (120 kVp) and the images resulting from studied reconstruction techniques: dual energy CT virtual monochromatic images at the 70 and 130 keV levels (70 keV DECT and 130 keV DECT), the iterative metal artefact reduction algorithm (MAR) and their combinations (70 keV DECT MAR and 130 keV DECT MAR). In each case the images were sorted and given to the observers as determined by a ranking of the results in study A and B (see Supplementary Table A3). Accordingly, the images are here presented in the order they were displayed to the observers from left to right. The true contour is shown in red and each of the green contours was delineated by one of the five observers. Note that on the two left-most reconstructions of the spine and hip phantom, not all observers detected the objects, hence these images contain less than five green contours.

Fig. 4 shows the observer contours of the low-contrast target in each reconstruction of the phantoms. 70 keV DECT images were not included in this study, as these images were ranked lower than the standard-of-care in study A and B. The structures delineated based on the ‘Metal Removed’ image (rightmost column in Fig. 4) were used as reference for the analysis.

*Dental filling phantom:* 130 keV DECT MAR images yielded the most accurate contours, reducing HD median by 63%, and increasing DC by 117%.

*Spine implant phantom:* 130 keV DECT images yielded the most accurate contours, reducing HD median by 47% and increasing DC median by 127%.

As seen in Fig. 4, the low-contrast object in the spine phantom had two sections in some slices, as opposed to the low-contrast target in the two other phantoms. No observers noticed the smallest section of the low-contrast object in the standard-of-care image, while one observer noticed it in the MAR reconstructions and three observers noticed it in the 130 keV DECT images.

*Hip implant phantom:* 130 keV DECT MAR images yielded the most accurate contours, reducing the HD median by 62%, and increasing the DC median by 176%.

A visual evaluation showed a clear improvement in delineation accuracy on the final three reconstructions in all phantoms. In addition, the Friedman and post hoc Nemenyi test revealed that the HD and DC values (see Table 2) in the standard-of-care images were significantly different from those found in the image without metal in all cases except one (DC for the spine phantom). However, the HD and DC metrics for the three best reconstructions in each phantom were not significantly different from the delineation metrics in the reference image.

**Table 2:** Median and range {Med [min–max]} of measured Hausdorff Maximum Distances and Dice’s Coefficients for the five contours performed by the observers and displayed in Fig. 4. An asterisk indicates *p* < 0.05 in the Friedman Analysis, indicating a result that shows a statistically significant difference to the values in the “Metal Removed” reference contours. In each case the modality resulting in the most accurate contours is highlighted with bold. Note, that some of the modalities produce quite similar results and in each phantom the three most accurate sets of observer contours are not significantly different from the reference contours.

Delineation Accuracy Metrics						
	120 kVp	130 keV DECT	70 keV DECT MAR	120 kVp MAR	130 keV DECT MAR	Metal Removed
*Hausdorff Maximum Distances [mm]*						
Dental	10.7 [6.3–14.5]^*^	9.8 [5.6–11.5]^*^	7.7 [4.1–9.2]	5.1 [3.9–8.6]	**4.0 [2.9**–**8.1]**	2.9 [2.0–3.3]
Spine	18.2 [17.8–22.0]^*^	**9.7 [7.9**–**18.6]**	18.7 [11.1–21.6]^*^	18.6 [10.4–19.1]	11.8 [10.4–16.9]	2.2 [2.0–3.1]
Hip	7.7 [5.3–10.2]^*^	6.2 [1.8–6.9]	3.6 [2.2–4.1]	3.5 [2.2–3.6]	**2.9 [2.8**–**5.0]**	2.0 [1.4–2.2]

*Dice’s Coefficient*						
Dental	0.75 [0.72–0.81]^*^	0.78 [0.75–0.80]^*^	0.84 [0.80–0.85]	0.87 [0.86–0.88]	**0.88 [0.83**–**0.89]**	0.91 [0.89–0.92]
Spine	0.57 [0.25–0.75]	0.69 [0.62–0.77]	0.48 [0.28–0.67]^*^	**0.74 [0.52**–**0.80]**	0.58 [0.53–0.72]	0.88 [0.87–0.91]
Hip	0.5 [0.31–0.73]^*^	0.60 [0.38–0.82]^*^	0.88 [0.70–0.91]	0.87 [0.85–0.91]	**0.88 [0.87**–**0.91]**	0.95 [0.92–0.96]

## Discussion

In this work, we evaluated six reconstructions from the perspective of accurate HU-value representation, preference of clinical observers in patient images and delineation accuracy. To the best of our knowledge, this is the first study to identify the optimal use of DECT and MAR techniques in clinical routine for treatment planning CTs and to quantify the gain in terms of delineation accuracy. While 130 keV DECT and MAR reconstructions showed different benefits and draw-backs, it was a consistent pattern in all three studies, that combined 130 keV DECT MAR reconstructions can be considered an ideal option for metal artefact reduction in general.

One concern when using MAR techniques is the use of linear interpolation to correct HU-values in artefact areas, leaving blurred areas, potentially making soft tissue borders inseparable as a result. Such an effect could pose a risk of missing important target volumes and failing to accurately identify organs at risk. This study confirms, however, that while the studied techniques do not create a perfect result, they do improve HU-value representation (as also shown by Axente et al. [28]) and soft tissue visibility in the artefact region. In addition, the techniques resulted in more accurate target delineation in clinically realistic phantoms. As target and OAR delineation variability is likely the largest source of error in radiotherapy, these results indicate a need for further study of delineation accuracy with MAR and DECT reconstructions through measurement of delineation inter-observer variability on treatment planning CTs from larger cohorts of patients referred for radiotherapy. Investigating the impact of these DECT and MAR techniques on radiotherapy dose calculation is also a topic of further study; however, the improved HU-value representation indicates that dose calculation accuracy may improve as well.

The technique of choice for artefact reduction varied by metallic implant type (Table 2 and Table A3). For patients with dental fillings, 130 keV DECT MAR images were found to be optimal in all three studies. For patients with spine implants, the results were mixed between different metrics, but the optimal reconstructions appeared to be 130 keV DECT and 130 keV DECT MAR. Specifically for spine implants, 130 keV DECT produced significantly better results than 120 kVp MAR. This finding adds new information to the study by Kuchenbecker et al. [29], in which dedicated MAR outperformed DECT. Our results support the implication in their work, that strong metal artefacts (i.e. typically artefacts from dental fillings and hip implants) are not removable with DECT. For patients with hip implants, 130 keV DECT MAR images performed best in the majority of metrics in study A and C, but 120 kVp MAR was preferred by the observers. This effect was likely caused by the relatively small area of the beam hardening artefact in the hip implant images.

One strength of this study is that images were ranked based on pairwise combinations of the reconstructions presented in random order. This allowed the observers to compare several distinct anatomical regions, such as bone edges and different soft tissues in all slices of both images, allowing for a more informed relative ranking of all reconstruction methods. Additionally, we eliminated potential bias which could have arisen from the order in which images were evaluated. Other studies have used a qualitative scale to evaluate and rank image quality [28], [21], which may limit the information on relative image quality (e.g. if the scale lacks discriminative power).

Under some circumstances, such as in a study by Riegel et al. [30], a reduction of delineation volume is considered an improvement, as this reduces the risk of treatment side-effects. However, in the present study we do not measure the tumour volume, based on reasoning clearly illustrated in Fig. 4: In several cases, the volume of the observer contours (green) are smaller than the true contour (red). Hence, volume reduction should not be considered an improvement.

One limitation of this study is the use of a single source scanner, which required that the patient was scanned twice for the DECT scan, once for each energy. A dual source scanner would be superior for clinical applications; however, the bore size of current dual source scanners is too small for some radiotherapy set-ups. Though this was not explicitly tested, careful visual evaluation did not reveal artefacts caused by shift in position. In general, it is a limitation of DECT techniques that the decision of whether or not to obtain a DECT scan must be made prior to the scan – in contrast, it is a benefit of the MAR technique that these reconstructions can be created based on raw-data from the standard-of-care scans. Hence, to make a decision about whether or not to use DECT, information about patient implants should be acquired prior to treatment planning CT scan acquisition. In addition, for dose calculation on DECT VM images, the treatment planning system HU-value-electron density curves must be specifically calibrated for each desired VM energy with an electron density phantom. These factors make DECT more cumbersome to implement than the MAR algorithm. However, the results of this study suggest that DECT 130 keV is preferable to MAR in presence of spine implants, and that combined DECT and MAR is the preferred modality in some cases for dental and hip implants. To optimize metal artefact reduction in the presence of all types of metallic implants, the implementation of DECT VM images for treatment planning should be considered.

DECT VM and MAR techniques improved the accuracy of HU-value representation and had a positive, statistically significant influence on observers’ preference. In addition, the techniques significantly improved delineation accuracy, and reduced delineation variability between observers. The artefact reduction technique of choice depends on the type of metallic implant, and a software environment where multiple reconstructions can be evaluated simultaneously is recommended.

## Conflict of interest statement

MA acknowledges the financial support of Cancer Research UK (Grant No. C8225/A21133).

## Funding sources

This study was partially financed by Siemens Healthineers and Varian Medical Systems in the form of a research grant to the department of Oncology at Rigshospitalet as well as an internal research grant from “Onkologisk Forskningsfond”, an internal oncology research foundation. These funding sources had no role in the acquisition, analysis and interpretation of the data, in the writing of the manuscript, or in the decision to submit the manuscript for publication.

## Footnotes

1 The maximum undirected Euclidean distance $d_{H}(X\text{,}Y)=\max\{{\overset{\to }{d}}_{H}(X\text{,}Y)\text{,}{\overset{\to }{d}}_{H}(Y\text{,}X)\}$ where ${\overset{\to }{d}}_{H}(X\text{,}Y)={\text{max}}_{x\in X}{\text{min}}_{y\in Y}d(x\text{,}y)$.

2 Dice’s similarity coefficient = 2|X∩Y|/(|X|+|Y|).

3 Note: The improvement in correct pixel representation also depends on the initial severity of the artefact in the image. The very heavy artefact which is produced by the hip implant causes the effect of this reconstruction to be much larger than with the dental and spine phantoms.
