# Supplementary material for: Metal artefact reduction for accurate tumour delineation in radiotherapy
Source: Radiother Oncol. 2018 Mar;126(3):479–86. doi: 10.1016/j.radonc.2017.09.029 (PMC5864514; doi:10.1016/j.radonc.2017.09.029)
Supplement: Supplementary data 1 [file mmc1.docx]

Supplementary Material

**Image Acquisition**

Standard CT and DECT images were acquired during one session on a 64-slice single-source CT scanner (Siemens Somatom Definition AS, Siemens Health Care, Forchheim Germany). The procedure was the following for all cases: images for RT planning were acquired at 120 kVp, and reconstructed using a B30f smooth convolution kernel for the hip and spine and H31s for the dental cases, while DECT scans were acquired at 80 kVp and 140 kVp and reconstructed using a D30f dual energy medium-soft convolution kernel for hip and spine and D34f for the head scan. The scans were acquired with a slice thickness of two millimetres and it was ensured that standard CT and DECT scan slices were aligned.

The resulting three scans at 120kVp, 80 kVp and 140 kVp were reconstructed using the MAR dental, MAR spine and the MAR hip algorithms respectively, (iMAR, Siemens AG, München Germany) depending on the type of implant examined. Moreover, the 80 kVp and 140 kVp image sets were combined using the Dual Energy application (syngo.CT Monoenergetic, Siemens AG) in order to calculate VM image reconstructions at 70 keV DECT and 130 keV DECT. Additional VM images at 70 keV DECT and 130 keV DECT were calculated based on MAR reconstructions of the 80 kVp and 140 kVp images, yielding VM MAR images.

In summary, this process resulted in six reconstructions of each phantom or patient: 1) 120 kVp (standard-of-care) 2) 120 kVp MAR 3) 70 keV DECT 4) 70 keV DECT MAR 5) 130 keV DECT and 6) 130 keV DECT MAR. Further details on parameters used for image acquisition and reconstruction are provided in supplementary table A1.

Table A1 In all cases the iterative metal artefact reduction algorithm (MAR) setting was selected according to the type of implant (dental MAR for the dental filling, etc.). The CareDose functionality was turned on during acquisition of standard clinical patient scans.

| **Key Acquisition Parameters** | | | | | | | | | | |
| --- | --- | --- | --- | --- | --- | --- | --- | --- | --- | --- |
| **Phantom** | Dental | | | Hip | | | Spine | | | |
| Scan number | 1 | 2 | 3 | 1 | 2 | 3 | 1 | 2 | 3 |  |
| Tube voltage [kVp] | 120 | 80 | 140 | 120 | 80 | 140 | 120 | 80 | 140 |  |
| Tube current [mA] | 160 | 330 | 157 | 314 | 344 | 166 | 314 | 474 | 113 |  |
| Convolution kernel | B31s | D30f | D30f | B31f | D30f | D30f | B31f | D30f | D30f |  |
| CTDIvol [mGy] | 16.5 | 7.4 | 8.5 | 15.9 | 7.0 | 8.2 | 15.9 | 7.3 | 8.4 |  |
| Spiral Pitch Factor | 0.8 | 0.55 | 1.1 | 0.8 | 0.6 | 1.2 | 0.8 | 0.8 | 0.8 |  |
| **Patients** | Dental | | | Hip | | | Spine | | | |
| Scan number | 1 | 2 | 3 | 1 | 2 | 3 | 1 | 2 | 3 |  |
| Tube voltage [kVp] | 120 | 80 | 140 | 120 | 80 | 140 | 120 | 80 | 140 |  |
| Tube current [mA] | 149 | 358 | 86 | 243 | 396 | 94 | 618 | 642 | 258 |  |
| Convolution kernel | H31s | D34f | D34f | B31f | D30f | D30f | B31f | D30f | D30f |  |
| CTDIvol [mGy] | 45.2 | 16.9 | 18.0 | 12.3 | 6.1 | 7.0 | 31.3 | 9.9 | 19.2 |  |
| Spiral Pitch Factor | 0.55 | 0.6 | 0.6 | 0.8 | 0.8 | 0.8 | 0.8 | 0.8 | 0.8 |  |

| **Artefact Quantification (Study A)** | | | | | |
| --- | --- | --- | --- | --- | --- |
| **Phantom** | **Reconstruction** | **Area**  **[mm^2]^** | **Severity**  **HU median and [IQR]** | **Water**  **[%]** | **Low-contrast target**  **[%]** |
| **Dental** | 120 kVp | 1874 | 151 (127) | 73 | 59 |
|  | 70 keV DECT | 1970 | 172 (140) | 72 | 56 |
|  | 130 keV DECT | 1657 | 144 (134) | 75 | 66 |
|  | 120 kVp MAR | 470 | 112 (54) | 97 | 98 |
|  | 70 keV DECT MAR | 561 | 134 (64) | 97 | 97 |
|  | 130 keV DECT MAR | 449 | 110 (39) | 95 | 97 |
| **Spine** | 120 kVp | 5363 | 98 (153) | 30 | 35 |
|  | 70 keV DECT | 5867 | 123 (218) | 26 | 24 |
|  | 130 keV DECT | 2814 | 177 (73) | 79 | 79 |
|  | 120 kVp MAR | 4182 | 71 (65) | 58 | 48 |
|  | 70 keV DECT MAR | 4420 | 87 (113) | 44 | 34 |
|  | 130 keV DECT MAR | 2798 | 123 (76) | 66 | 75 |
| **Hip** | 120 kVp | 6460 | 256 (376) | 3 | 4 |
|  | 70 keV DECT | 6593 | 262 (387) | 1 | 3 |
|  | 130 keV DECT | 5869 | 225 (276) | 13 | 9 |
|  | 120 kVp MAR | 3531 | 50 (94) | 50 | 64 |
|  | 70 keV DECT MAR | 4709 | 53 (86) | 31 | 46 |
|  | 130 keV DECT MAR | 2586 | 81 (80) | 65 | 86 |

Table A2 Analysis results of the phantom image analysis using dual energy CT virtual monochromatic reconstructions (DECT VM) and iterative metal artefact reduction algorithms (MAR). The artefact area and severity [Hounsfield (HU) median and inter-quartile range (IQR)] are reported. The two right-most columns present the percentage of correctly represented pixels in water and low-contrast target.

| **Ranking of Reconstructions Based on Phantom Image Analysis and Preferred Reconstructions** | | | | | | |
| --- | --- | --- | --- | --- | --- | --- |
| **Dental** | Standard-of-care | 70 keV DECT | 130 keV DECT | 120 kVp MAR | 70 keV DECT MAR | 130 keV DECT MAR |
| Art Area | 5.0 | 6.0 | 4.0 | 2.0 | 3.0 | 1.0 |
| Severity | 5.0 | 6.0 | 4.0 | 2.0 | 3.0 | 1.0 |
| % Accurate repr. Water | 5.0 | 6.0 | 4.0 | 1.5 | 1.5 | 3.0 |
| % Accurate repr. Tumour | 5.0 | 6.0 | 4.0 | 1.0 | 2.5 | 2.5 |
| Clinician Image Ranking | **5.0** | **6.0** | 4.0 | 3.0 | 2.0 | 1.0 |
| **Average rank** | **5.0** | **6.0** | **4.0** | **1.9** | **2.4** | **1.7** |
| **Spine** | Standard-of-care | 70 keV DECT | 130 keV DECT | 120 kVp MAR | 70 keV DECT MAR | 130 keV DECT MAR |
| Art Area | 5.0 | 6.0 | 2.0 | 3.0 | 4.0 | 1.0 |
| Severity | 5.0 | 6.0 | 3.0 | 1.0 | 4.0 | 2.0 |
| % Accurate repr. Water | 5.0 | 6.0 | 1.0 | 3.0 | 4.0 | 2.0 |
| % Accurate repr. Tumour | 4.0 | 6.0 | 1.0 | 3.0 | 5.0 | 2.0 |
| Clinician Image Ranking | 4.0 | 6.0 | 2.0 | 3.0 | 5.0 | 1.0 |
| **Average rank** | **4.6** | **6.0** | **1.8** | **2.6** | **4.4** | **1.6** |
| **Hip** | Standard-of-care | 70 keV DECT | 130 keV DECT | 120 kVp MAR | 70 keV DECT MAR | 130 keV DECT MAR |
| Art Area | 5.0 | 6.0 | 4.0 | 2.0 | 3.0 | 1.0 |
| Severity | 5.0 | 6.0 | 4.0 | 1.0 | 2.0 | 3.0 |
| % Accurate repr. Water | 5.0 | 6.0 | 4.0 | 2.0 | 3.0 | 1.0 |
| % Accurate repr. Tumour | 5.0 | 6.0 | 4.0 | 2.0 | 3.0 | 1.0 |
| Clinician Image Ranking | 4.0 | 6.0 | 5.0 | 1.0 | 2.0 | 3.0 |
| **Average rank** | **4.8** | **6.0** | **4.2** | **1.6** | **2.6** | **1.8** |

Table A3 Ranking of the image quality (1: best, 6: worst) based on the artefact quantification (study A) and pairwise ranking by oncology clinician (Study B). The order of presentation in the study of delineation accuracy (study C) presented in the bottom three rows was based on the average rank. Combined 70 keV dual energy CT virtual monochromatic (DECT VM) images were not included in this part of the study, as they were ranked worse than the standard-of-care in all cases of study A as well as B.
